# Fatal West Nile Encephalomyelitis in a Young Woman with Hypoparathyroidism and Sjögren’s Syndrome. Molecular Insights into Viral Neuro-Invasivity

**DOI:** 10.3390/ijms27010104

**Published:** 2025-12-22

**Authors:** Pasquale Padalino, Laura Secco, Eva Grosso, Giorgia Franchetti, Stefano Palumbi, Renzo Giordano, Guido Viel

**Affiliations:** Legal Medicine and Toxicology, Department of Cardiac, Thoracic, Vascular Sciences and Public Health, University of Padova, Via G. Falloppio 50, 35121 Padova, Italy; pasquale.padalino@studenti.unipd.it (P.P.); laura.secco@studenti.unipd.it (L.S.); eva.grosso@studenti.unipd.it (E.G.); giorgia.franchetti@unipd.it (G.F.); stefano.palumbi@studenti.unipd.it (S.P.); renzo.giordano.padova@gmail.com (R.G.)

**Keywords:** west Nile virus, WNV, viral encephalomyelitis

## Abstract

West Nile virus (WNV) is an arthropod-borne flavivirus first identified in 1937. Over time, WNV has spread globally and is now endemic in Italy. Although most human WNV infections are asymptomatic (80%), less than 1% progress to a neuroinvasive disease with high mortality rates. This case involves a 45-year-old woman with post-surgical hypoparathyroidism and Sjögren’s syndrome who developed severe encephalomyelitis linked to WNV, leading to ventilator-associated pneumonia and death. Neuropathological findings revealed a bilaterally cribriform thalamus and reddish punctate lesions near the dentate nucleus of the cerebellum. The trachea and bronchial hilum branches contained whitish foamy liquid. The left lung showed multiple brownish-violet areas, with whitish regions at dissection. The heart appeared unremarkable. A detailed neuropathological examination focused on areas involved in motor control pathways. Tissue samples were stained with hematoxylin and eosin and trichrome techniques, and immunohistochemistry was performed using CD68, CD3, and CD20. A significant damage was observed in the lenticular nucleus and motor thalamus, with prominent concentric vascular calcifications. The cerebellar cortex showed near-total depletion of Purkinje cells. In the spinal cord, CD68 and CD3 positivity was noted in the lateral funiculi, anterior horns, and Clarke’s column. Lung findings showed pulmonary edema, chronic emphysema, and bronchopneumonia. The observed CD3 and CD68 positivity confirms that WNV spreads trans-synaptically along motor control pathways. We speculate on the potential molecular mechanisms by which hypoparathyroidism and Sjögren’s syndrome may have played a role in the neuroinvasive progression of the disease.

## 1. Introduction

West Nile virus (WNV) is an arthropod-borne flavivirus that was first isolated from the blood of a febrile Ugandan woman in 1937 [1]. Like other flaviviruses, it is a small, enveloped virus containing a single positive-sense RNA genome [2]. Eight phylogenetic lineages are currently known: Lineage 1 (L1) to Lineage 8 (L8), with WNV L1 and L2 being the most widespread and pathogenic ones [3].

The virus exhibits a roughly spherical morphology, with an approximate diameter of 50 nm, composed of an icosahedral nucleocapsid enclosed within a lipid envelope [4,5]. Its genomic RNA, approximately 11 kilobases (kb) in length, encodes a single long open reading frame (ORF) flanked by two non-coding regions [2]. The ORF is translated into a polyprotein, which is subsequently cleaved by both cellular and viral proteases into three structural proteins—capsid, envelope, and pre-membrane—and seven nonstructural proteins (NS1, NS2A, NS2B, NS3, NS4A, NS4B, and NS5) [5,6]. Among the structural proteins, the envelope protein plays a critical role in binding to cellular receptors, mediating membrane fusion, and facilitating viral entry into host cells. The nonstructural proteins, on their part, are essential for viral genome replication, virion assembly, and pathogenesis [7]. For example, NS1 has been shown to modulate host immune responses by inhibiting complement activation and downregulating the interferon (IFN) response [5].

WNV is predominantly transmitted through biological vectors, with Culex mosquitoes identified as the primary competent ones [8,9,10]. These mosquitoes acquire the virus during a blood meal from a viremic vertebrate host, with birds being the predominant and preferred reservoir [8,11]. Following ingestion of the blood meal, WNV reaches the mosquito midgut, where the virus is amplified and subsequently spreads to the salivary glands [12,13]. When an infected mosquito feeds on humans or other animals, WNV may be inoculated into the host’s skin, where it infects keratinocytes and Langerhans cells. These cells migrate to regional lymph nodes, where initial viral replication occurs [14]. WNV then spreads systemically to visceral organs such as the kidney and spleen, where a second round of replication presumably takes place in epithelial cells and macrophages, respectively [8,15]. The blood viral load of an infected mammal is usually not as high as in birds and is therefore insufficient to infect another mosquito; thus, humans and other animals are considered dead-end hosts in the WNV transmission cycle [16]. However, blood transfusions and organ transplants from previously infected individuals represent additional sources of WNV infection [17].

Infected individuals exhibit no symptoms in approximately 80% of cases. Among those who do develop symptoms, the illness typically manifests as a febrile syndrome, with fewer than 1% progressing to neuroinvasive disease, such as encephalitis. In severe cases, the disease may lead to acute flaccid paralysis following meningitis or encephalitis, characterized by rapidly worsening symptoms that can affect all four limbs [18].

The mechanism of WNV neuroinvasion has been debated for years, with two main routes receiving the most attention: haematogenous and transneural. Various mechanisms have been proposed for both pathways [17]. The envelope (E) glycoprotein of WNV has been implicated in neuroinvasiveness, particularly domain III of the protein, which constitutes the receptor binding domain. The haematogenous route suggests that WNV enters the central nervous system (CNS) through the bloodstream, crossing either endothelial or choroid plexus epithelial cells, or by passive movement caused by increased vascular permeability. In contrast, the transneural route proposes that the virus spreads along sensory and motor nerves via direct or retrograde axonal transport from infected peripheral neurons, gaining access to the CNS through peripheral or olfactory nerves by infecting olfactory neurons and spreading to the olfactory bulb. Other proposed mechanisms include passive migration of free virus particles across a disrupted blood–brain barrier and the “Trojan Horse” mechanism, where infected leukocytes carry the virus into the CNS [8,17].

Immunocompromission (i.e., HIV or recipient of organ transplant) is the main risk factor for developing West Nile Neuroinvasive Disease (WNND) after infection with WNV [19]. Additionally, increasing age, associated with impairments in innate immunity and age-related defects in T cell-mediated immunity, also increases the risk of WNND [20].

In this paper, we present the case of a 45-year-old woman with post-surgical hypoparathyroidism and Sjögren’s syndrome who developed severe West Nile virus-associated encephalomyelitis, ultimately leading to her death due to complications from mechanical ventilation. We explore in detail the progression of her condition, emphasizing how the interplay of these pre-existing conditions may have contributed to the development of WNND, an association not reported in previous scientific studies.

## 2. Results

### 2.1. Case History

A 45-year-old female, presenting with a febrile condition for approximately four days, had initiated antibiotic therapy upon her primary care physician’s recommendation. She was found unconscious at home and subsequently transported to the Emergency Department, where laboratory and instrumental investigations were performed, including cranial and brain CT, angio-CT of the epicardiac vessels, brain MRI, EEG, and cerebrospinal fluid analysis, allowing the exclusion of acute ischemic events, seizures, and bacterial meningitis.

On the suspect of viral encephalitis, the patient was admitted to the Neurology department, where both empirical antibiotic and antiviral treatments were initiated. On the first day of hospitalization, the patient was alert and responsive to various clinical assessments. However, her condition progressively worsened over the following days, with the development of clonic movements, dysphagia, and hemodynamic instability, prompting a transfer to the intensive care unit.

Upon transfer, the patient was noted to be in a soporose state, mute, unable to follow simple commands. At admission, the patient exhibited severe respiratory distress, leading to oro-tracheal intubation and continuous mechanical ventilation under sedation.

A lumbar puncture for cerebrospinal fluid sampling allowed the diagnosis of West Nile Virus infection through the detection of anti–West Nile Virus IgM antibodies through chemiluminescence. Viral RNA was subsequently detected in the blood by qualitative real-time PCR. In response, the patient was treated with prednisone, given the lack of specific antiviral therapy for WNV. In the subsequent days, a surgical tracheostomy was performed.

Due to the onset of focal seizures and fever, blood and urine cultures were performed, with the last one yielding positive for *Pseudomonas aeruginosa*, prompting the initiation of antibiotic therapy. Days later, a chest X-ray revealed a thin layer of pneumothorax on the left side, as well as an evident consolidation in the retrocardiac area. A bronchoaspirate culture was performed, which remained positive for *Pseudomonas aeruginosa*.

In the following days, the clinical condition remained stable, with occasional hyperpyrexia due to the microorganism’s resistance to the antibiotics used. The patient was then transferred back to the Neurology Department and placed in isolation.

After a month, with clinical stabilization, the patient was transferred to a nursing home. Over the subsequent days, the patient underwent regular tracheo-bronchial aspirations and dressings for multiple pressure ulcers.

The patient remained stable at the facility until, upon the onset of dyspnea, the emergency services were contacted, and she was brought to the Emergency Department.

In the Emergency Department, the patient underwent laboratory tests, urinalysis, arterial blood gas analysis, ECG, chest X-ray, and abdominal X-ray. She was then transferred to the Internal Medicine Unit.

In the following days, the patient was placed in isolation due to a bronchoaspirate culture positive for multi-resistant *Pseudomonas aeruginosa*.

The patient remained clinically stable in the following days. However, approximately 10 days after admission, the attending physicians found the patient bradyapneic, with a saturation of 33%. An arterial blood gas (ABG) revealed respiratory failure with severe acidosis. A pulmonologist was contacted and attempted to modify the settings and mode of the mechanical ventilator. Despite these efforts, the patient died due to respiratory arrest.

### 2.2. Autopsy Findings

A forensic autopsy was performed on behalf of the public prosecutor; the aim of the exam was to determine the cause of death and to investigate any potential professional liability of the physicians involved in the case management.

The external examination revealed only signs of acupuncture, tracheostomy, and the percutaneous endoscopic gastrostomy (PEG) performed during hospitalization.

During the autopsy, the examination of the cranial cavity showed no significant macroscopic alterations, and the brain was entirely removed after formalin fixation. In the thoracic cavity, regarding the respiratory system, the trachea contained a small amount of whitish foamy liquid material, which was also found within the bronchial hilum branches. The left lung exhibited multiple areas of brownish-violet color upon inspection and diffused whitish areas upon sectioning. The heart was entirely removed for examination after formalin fixation.

In the abdomen, the gastric mucosa appeared hyperemic, with some red petechial spots, and coated with a semi-liquid yellowish material, identical to that found in the esophagus. The liver, spleen, intestines, and kidneys were unremarkable.

After removal of the abdominal viscera, the T10 and L2 vertebrae were first transversely sectioned, followed by a coronal section of the entire thoracolumbar vertebral segment between these two vertebrae, anterior to the transverse processes. Removal of the anterior portion of the vertebral arch allowed access to the spinal canal, revealing the dura mater, which appeared white-pearlescent in color. The observed segment was then removed for examination after fixation in formalin.

The main thoracic and abdominal arterial vessels showed scattered calcific plaques.

### 2.3. Examination After Formalin Fixation

The examination of the heart following fixation in formalin did not reveal any remarkable pathological findings.

Regarding the brain, upon palpation, a reduced consistency was observed in the area of the olfactory bulbs. In the coronal sections of the brain parenchyma, the thalamus exhibited a bilaterally cribriform appearance. Near the dentate nucleus of the cerebellum, there was a presence of reddish punctate lesions.

No significant macroscopic alterations were noted in the harvested spinal cord, although upon sectioning, the anterior horns appeared poorly defined.

### 2.4. Histological Findings

The examination of the frontal and temporal cortices revealed no significant lesions.

**Figure 1:**
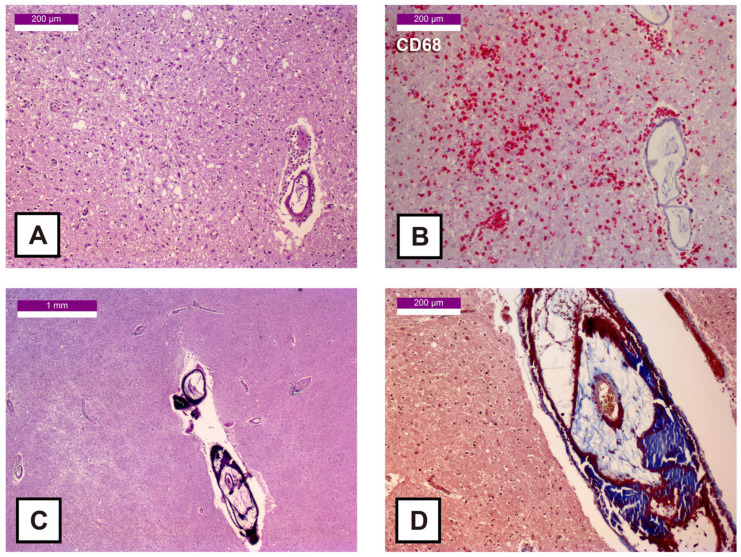
Histological samples of the lenticular nucleus: (**A**) 100× HE stain—Cerebral edema with neuronal loss, accompanied by astrocytic hypertrophy and hyperplasia; (**B**) 100× CD68 stain (Fast Red)—Microglial activation with macrophagic proliferation; (**C**) 25× HE stain—Neuronal necrosis and vascular calcifications; (**D**) 100× Masson’s trichrome stain—Concentric vascular calcification with luminal narrowing.

**Figure 2:**
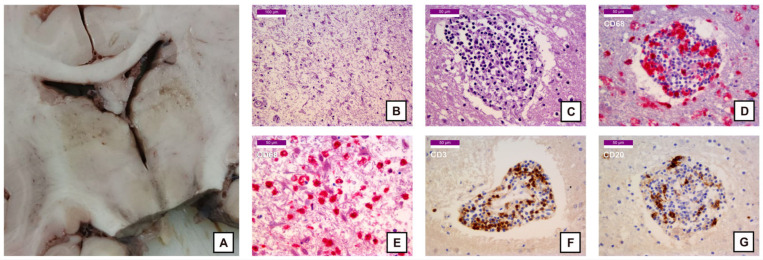
Pictures of the thalamus and histological samples: (**A**) Thalamus (macroscopic view) showing a cribriform appearance of the motor thalamus; (**B**) 200× HE stain—Marked parenchymal rarefaction with astrogliosis; (**C**) 400× HE stain—Perivascular cuffing by round cells; (**D**) 400× CD68 stain (Fast Red)—Perivascular cuffing composed of many monocytes and macrophages, also infiltrating the surrounding parenchyma; (**E**) 400× CD68 stain (Fast Red)—Parenchymal rarefaction with macrophagic proliferation; (**F**) 400× CD3 stain (DAB)—Perivascular cuffing predominantly composed of T lymphocytes; (**G**) 400× CD20 stain (DAB)—Perivascular cuffing with a minor B lymphocyte component.

However, considerable areas of damage were observed in the lenticular nucleus and in the motor thalamus. In both areas, a marked parenchymal rarefaction consistent with neuronal necrosis, astrogliosis and lymphocytic inflammatory infiltrates with perivascular cuffing were noted (Figure 1 and Figure 2). Immunohistochemistry for CD68 showed widespread positivity in the gray matter (Figure 1B and Figure 2D,E), indicating microglial activation (i.e., rounder and larger cell body with reduced arborization and complexity of ramified processes) and macrophage proliferation. Notably, the lenticular nucleus displayed an unusually prominent concentric vascular calcification, despite the patient’s young age (Figure 1C,D). As evidenced by images of the thalamus, the lymphocytic infiltrate (Figure 2C) was predominantly CD3+ (Figure 2F), although a CD20+ component was also present (Figure 2G). Histological examination of the hippocampus showed only mild CD3+ infiltrates, with preserved architecture and no evidence of neuronal loss.

**Figure 3:**
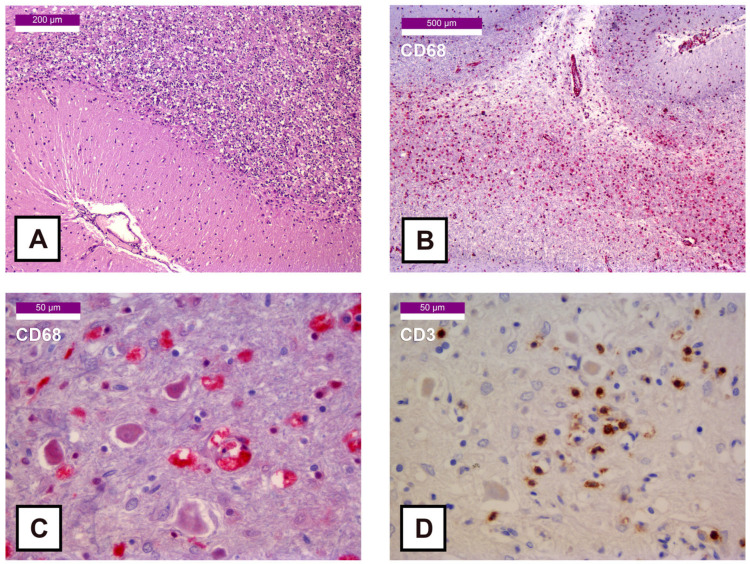
Histological samples of the cerebellar cortex: (**A**) 100× HE stain—Cerebellar cortex with near-complete loss of Purkinje cells; (**B**) 50× CD68 stain (Fast Red)—Subcortical white matter with necrosis and prominent macrophagic infiltration; (**C**) 400× CD68 stain (Fast Red)—Macrophagic infiltration with degenerating neurons; (**D**) 400× CD3 stain (DAB)—T lymphocyte infiltration.

The images of the cerebellar cortex revealed almost complete depletion of Purkinje cells (Figure 3A), with marked CD68 positivity in the white matter (Figure 3B). In contrast to the more extensive damage observed in the thalamus, the dentate nucleus showed a less pronounced injury, yet it displayed an infiltrate positive for CD68 (Figure 3C) and CD3 (Figure 3D), with no positivity for CD20. The Perls’ Prussian Blue staining performed on the dentate nucleus did not reveal significant hemosiderin deposits or additional pathological findings.

**Figure 4:**
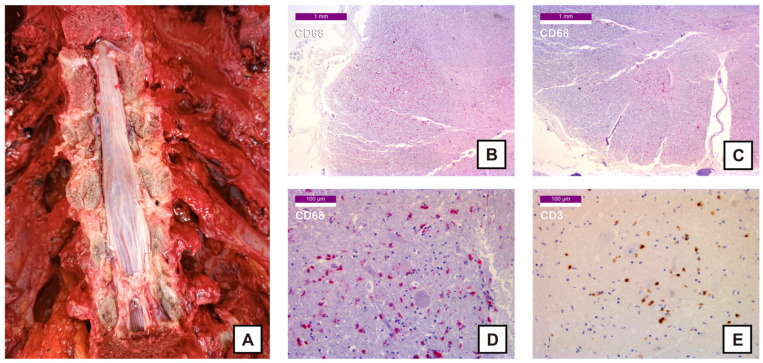
Pictures of the spinal cord and histological samples: (**A**) Spinal cord (macroscopic view) following removal of the vertebral bodies; (**B**) 25× CD68 stain (Fast Red)—Macrophagic infiltration of the lateral columns and Clarke’s column; (**C**) 25× CD68 stain (Fast Red)—Macrophagic infiltration of the anterior horns; (**D**) 200× CD68 stain (Fast Red)—Degenerating neurons with neuronal shrinkage and neuronal swelling in the anterior horns; (**E**) 200× CD3 stain (DAB)—T lymphocyte infiltration of the anterior horns.

Spinal cord sections (Figure 4A) revealed CD68 positivity in the lateral funiculi within the white matter and in the anterior horns within the gray matter (Figure 4B–D), with notable positivity also observed in the Clarke’s column. In the anterior horns CD3 positivity was also apparent (Figure 4E) with degenerating neurons manifesting neuronal shrinkage and neuronal swelling. Immunohistochemical staining for S100B did not yield additional or diagnostically significant findings.

**Figure 5:**
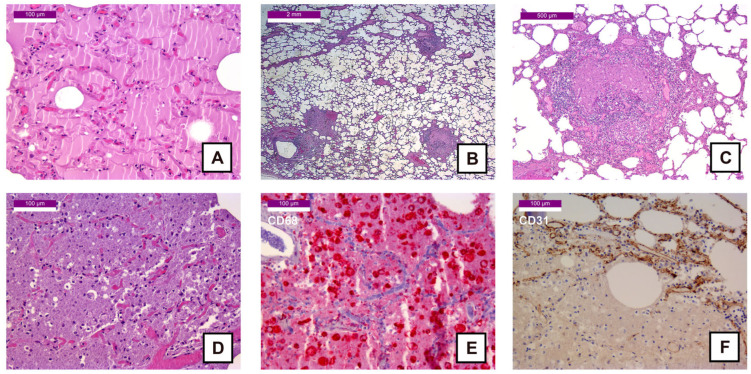
Histological samples of the lung: (**A**) 200× HE stain—Pulmonary edema; (**B**) 12.5× HE stain—Pulmonary emphysema with bronchiolitis; (**C**) 50× HE stain—Bronchiolitis with lymphocytic wall infiltration and intraluminal cellular debris; (**D**) 200× HE stain—Intra-alveolar cellular debris as sequelae of bronchopneumonia; (**E**) 200× CD68 stain (Fast Red)—Intra-alveolar macrophagic infiltration; (**F**) 200× CD31 stain (DAB)—Alveolar wall destruction with loss of the capillary network.

The lungs exhibited features consistent with pulmonary edema (Figure 5A,C) and chronic emphysema (Figure 5B), as well as sequelae of bronchopneumonia, with alveoli filled with necrotic material positive for CD68 (Figure 5D,E) and endothelium negative for CD31 in the affected areas (Figure 5F). Additionally, there was evidence of chronic obstructive bronchiolitis, characterized by a significant peribronchiolar lymphocytic infiltrate and endoluminal necrotic material (Figure 5C) positive for CD68. The histochemical and immunohistochemical stainings performed with PAS, CK AE1/AE3, and calretinin did not provide additional diagnostically significant findings.

## 3. Discussion

Forensic autopsy followed by histological and immunohistochemical analyses revealed an inflammatory encephalomyelitis characterized by areas of neuronal necrosis and CD3+ and CD68+ immunohistochemical positivity in the thalamus, lenticular nucleus, cerebellar dentate nucleus, and within the spinal cord—specifically in the anterior horns, Clarke’s column, and lateral tracts.

CD3 is a protein complex that forms an integral part of the T-cell receptor (TCR). The primary function of CD3 is to transmit the signal from the external environment to the intracellular compartment of the T cell upon TCR activation by antigen binding. The CD3 chains contain Immunoreceptor Tyrosine-based Activation Motifs (ITAMs), which become phosphorylated upon TCR engagement and serve as docking sites for protein tyrosine kinases, such as ZAP-70, which are crucial for initiating signaling cascades that lead to T cell activation [21]. The presence of CD3 at all stages of T-cell development makes it a useful immunohistochemical marker for T cells in tissue sections. T cells are essential for acquired cell-mediated immunity, as they play a crucial role in killing cancer cells, cells infected by intracellular pathogens such as viruses, or cells that are damaged in other ways.

CD68 is a highly glycosylated type-I transmembrane glycoprotein that is mainly bound to the endosomal/lysosomal compartment but can rapidly translocate to the cell surface. The role of CD68 in inflammation and immunity is still a mystery, and its role as a scavenger receptor remains to be confirmed. [22]. As an immunohistochemical marker, it is particularly useful to identify the various cells of the macrophage lineage, including monocytes, histiocytes, giant cells, Kupffer cells, osteoclasts and microglia.

The identification of CD3+ T lymphocytes, alongside CD68+ microglia activation and macrophage proliferation, strongly suggests a T cell-mediated inflammatory response characteristic of viral-induced tissue damage, although further immunohistochemical analyses to more precisely differentiate lymphocyte T subtypes were not performed. This immunopathological profile indicates an initial adaptive immune reaction primarily driven by T cells targeting infected cells, followed by the activation of microglia and the recruitment of macrophages. Microglial activation is a key element of the neuroinflammatory response to WNV infection. Microglia respond to pathological changes in the CNS by becoming primed, that is, more susceptible to activation, and by adopting a morphology characterized by a rounder and larger cell body with reduced arborization and complexity of ramified processes [23,24]. While initially protective and essential for viral clearance, dysregulated T cell–driven microglial responses may contribute to neuronal injury and long-term impairment, persisting even after viral elimination from the CNS [25,26,27].

The observed localization of these inflammatory infiltrates in the thalamus, lenticular nucleus, cerebellar dentate nucleus, and within the spinal cord—specifically in the anterior horns, Clarke’s column, and lateral tracts—aligns with previous neuropathological findings in WNND [28,29,30]. Indeed, previous studies have demonstrated that West Nile Virus (WNV) spreads transsynaptically along motor control pathways [28]. Such movement of WNV particles is facilitated by microtubules [31]. Upon entry, WNV utilizes clathrin-mediated endocytosis to internalize the virus into the cells. Once inside the cell, the virus particles are trafficked along the endosomal and lysosomal pathways, involving the microtubule network. The viral structural proteins C and E interact with kinesin to promote this process [32]. A pH-dependent fusion event then occurs, releasing the viral nucleocapsid into the cytoplasm, near the endoplasmic reticulum [33].

Hippocampal involvement has also been reported in previous studies on WNV [34] and other viral [35] and non-viral encephalitis [36]; however, in the presented case, only mild CD3^+^ infiltration was observed, in the absence of neuronal loss. 

WNV-induced damage results from a combination of direct viral infection and indirect damage due to inflammatory responses. Astrocytes, which normally regulate glutamate levels, become dysfunctional during infection as observed in this case, where they exhibit pronounced hypertrophy and hyperplasia. This leads to a reduction in the expression of GLT-1/EAAT-2 and to an impaired glutamate uptake that causes an accumulation of glutamate in the extracellular space, which then damages both infected and uninfected motor neurons in the spinal cord. The resulting loss of motor neurons contributes to the clinical manifestation of acute flaccid paralysis [37].

The case reported here is emblematic because it describes the development of WNND in a 45-year-old woman, notably a younger age than the one typically reported in the Literature, with a sixfold higher risk in patients over 65 [38]. Her only comorbidities were Sjogren’s syndrome, treated with prednisone and mycophenolate, and postoperative hypoparathyroidism (HypoPTH), managed with oral calcium supplementation.

Although there is only limited evidence linking rheumatic diseases, such as Sjögren’s syndrome (SS), with WNND [39], the immunosuppressive therapy the patient was receiving may have contributed to the worsening of the infection; SS is also inherently associated with immune dysregulation, resulting in elevated local and systemic levels of pro-inflammatory cytokines [40]. Further associations between autoimmunity and viral encephalitis, specifically regarding HSV, have been described [41]. This inflammatory milieu may have contributed to an increased BBB permeability, thereby facilitating viral entry into the CNS.

Regarding postoperative HypoPTH, this is a clinical disorder caused by a deficiency of parathyroid hormone (PTH) following thyroid surgery, biochemically characterized by hypocalcemia and hyperphosphatemia [42]. Although there is no evidence in the literature supporting it as a risk factor for the development of WNND, clinically it typically presents with neuromuscular symptoms and ectopic soft tissue calcifications [43], sometimes even affecting cerebral structures, as evidenced in this specific case by the prominent concentric vascular calcifications observed in the lenticular nucleus [44]. Even before the molecular composition of the blood–brain barrier (BBB) was fully defined, fluctuating intracellular Ca^2^+ levels were known to affect BBB integrity. A key determinant in regulating paracellular permeability is the endothelial cytoplasmic Ca^2+^ concentration that affects junctional and cytoskeletal proteins [45]. An experimental study demonstrated that low extracellular Ca^2+^ induces a marked increase in brain endothelial permeability, mediated by intercellular Ca^2+^ waves (ICWs) through the involvement of gap junction channels and hemichannels [46]. Rapid changes in the endothelial cytoplasmic Ca^2+^ concentration may alter BBB permeability by disrupting adherens (AJ) and tight junctions (TJ) via different signal transduction pathways, such as the PKC cascade, or through direct interaction with junctional proteins [47,48]. It has also been reported a link between reduced serum calcium levels in patients with viral encephalitis, through the activation of Akt-mediated signaling pathway, which is a modulator of calcium influx, promoting viral entry [49].

Transendothelial migration across the blood–brain barrier (BBB) is indeed reported in the literature as a potential alternative route for WNV entry into the central nervous system (CNS). Therefore, in the presented case, an initial transendothelial spread across the BBB occurring in a context of increased BBB permeability potentially favored by calcium fluctuations related to hypoparathyroidism (and its treatment), as well as by the pro-inflammatory milieu associated with Sjögren’s syndrome, might have occurred. Once the virus reached the CNS, transaxonal dissemination through neurofunctionally connected regions appears consistent with the observed pattern of CNS involvement. These speculations represent the most plausible pathophysiological hypotheses to explain WNV neuroinvasiveness in the presented case, although further and more in-depth investigations are needed to elucidate the exact molecular behavior of WNV in WNND.

Regarding the cause of death, it was determined to be an acute on chronic respiratory failure secondary to a diffuse chronic bronchiolitis, in sequelae of *Pseudomonas aeruginosa* pneumonia associated with mechanical ventilation and sequelae of encephalomyelitis due to West Nile virus, as supported by the clinical history, the autopsy and the histopathological findings.

## 4. Materials and Methods

This case was assigned to the Institute of Legal Medicine of the University of Padua (Italy) by the judicial authority to investigate any hypotheses of professional liability. The case history was reconstructed based on the medical records and the information provided by the family members to the judicial authority.

The autopsy was performed in accordance with the European Guidelines on Forensic Autopsies [50] and the standards set by the European Council of Legal Medicine [51]. During autopsy, the heart and brain were entirely removed, as well as the T10–L2 segment of the spinal cord, and fixed in 10% buffered formalin. These organs were subsequently examined 30 days after formalin fixation. Specifically, the heart examination was conducted according to the Guidelines for Autopsy Investigation of Sudden Cardiac Death [52].

Additional tissue specimens were collected from the adrenal glands, liver, lungs, kidneys, pancreas, and spleen; all specimens were fixed in 10% buffered formalin, dehydrated, paraffin-embedded, and stained with Haematoxylin and Eosin (HE). For central nervous system sections, Masson’s trichrome, Perls’ Prussian blue, and immunohistochemical stains for CD3, CD20, CD68, and S100B were performed. In addition, Periodic acid–Schiff (PAS) and immunohistochemical stains for CD31, CK AE1/AE3 and calretinin were applied in lung sections. The preparation of histological slides was carried out in a certified laboratory under the supervision of a qualified pathologist, in accordance with routine histopathological protocols.

## 5. Conclusions

The presented case prompts further speculations on the molecular pathways that might contribute to the progression of West Nile virus (WNV) infection into West Nile neuroinvasive disease (WNND) in young patients with hypoparathyroidism and Sjögren’s syndrome. Although it is well-known that immunocompromission is the main causative factor for developing West Nile Neuroinvasive Disease (WNND), hypoparathyroidism and Sjögren’s syndrome have not yet been proposed as potential risk factors for WNND.

In this paper we explore some hypotheses that could connect these pre-existing conditions, and particularly the post-operative hypoparathyroidism, to the development of a fatal WNND, although further and more in-depth studies are needed to elucidate the exact pathogenic mechanisms behind this association.

Identifying risk factors and understanding them at the molecular level is essential for developing preventive strategies and therapies in cases of WNV infections in individuals predisposed to WNND.

## Data Availability

The original contributions presented in this study are included in the article. Further inquiries can be directed to the corresponding author.

## Abbreviations

The following abbreviations are used in this manuscript:

ABG	Arterial Blood Gas
AJ	Adherens Junction
BBB	Blood–Brain Barrier
C	Capsid protein
CD3	Cluster of Differentiation 3
CD20	Cluster of Differentiation 20
CD31	Cluster of Differentiation 31
CD68	Cluster of Differentiation 68
CKAE1	Cytokeratin AE1
CNS	Central Nervous System
CT	Computed Tomography
E	Envelope protein
EAAT-2	Excitatory Amino Acid Transporter 2
ECG	Electrocardiogram
EEG	Electroencephalogram
GLT-1	Glutamate Transporter 1
HE	Haematoxylin and Eosin
HypoPTH	Hypoparathyroidism
HIV	Human Immunodeficiency Virus
IFN	Interferon
ITAM	Immunoreceptor Tyrosine-based Activation Motifs
L1	Lineage 1
L2	Lineage 2
L8	Lineage 8
MRI	Magnetic Resonance Imaging
NS1	Non Structural protein 1
NS2A	Non Structural protein 2A
NS2B	Non Structural protein 2B
NS3	Non Structural protein 3
NS4A	Non Structural protein 4A
NS4B	Non Structural protein 4B
NS5	Non Structural protein 5
ORF	Open Reading Frame
PEG	Percutaneous Endoscopic Gastrostomy
PKC	Protein Kinase C
PTH	Parathormone
S100B	S100 calcium-binding protein B
SS	Sjögren Syndrome
TCR	T-cell Receptor
TJ	Tight Junction
WNND	West Nile Neuroinvasive Disease
WNV	West Nile Virus
ZAP70	ζ-Associated Protein of 70 kDa
